# Pain intensity and comorbid depressive symptoms in the general population: An analysis of the German Health Update Study (GEDA 2019/2020‐EHIS)

**DOI:** 10.1002/ejp.4745

**Published:** 2024-10-23

**Authors:** Jan Niklas Ahrend, Kathrin Jobski, Carsten Bantel, Falk Hoffmann

**Affiliations:** ^1^ Fakultät VI Medizin Und Gesundheitswissenschaften; Department für Versorgungsforschung, Bismarckstraße 20 Carl Von Ossietzky University Oldenburg Germany; ^2^ Fakultät VI Medizin Und Gesundheitswissenschaften; Department für Versorgungsforschung Carl Von Ossietzky Universität Oldenburg Oldenburg Germany; ^3^ Anesthesiology, Critical Care, Emergency Medicine, and Pain Management, School of Medicine and Health Sciences University of Oldenburg Oldenburg Germany

## Abstract

**Background:**

Pain and depressive symptoms often co‐occur, but the influence of pain intensity remains unclear. This study analyses the association between pain intensity and depressive symptoms in the general adult German population.

**Methods:**

Data was obtained from the cross‐sectional German Health Update Study (GEDA 2019/2020‐EHIS). Pain intensity in the last 4 weeks was categorized into no pain, mild, moderate, and severe. Depressive symptoms were assessed using the 8‐item Patient Health Questionnaire (PHQ‐8). The prevalence of depressive symptoms was analysed including a 95% confidence interval (95% CI). A multivariable logistic regression analysed associated factors with depressive symptoms by odds ratio (OR).

**Results:**

Of 22,708 participants (51.0% women, 35.1% aged 45–64 years), 41.2% reported no pain, 32.1% mild, 15.3% moderate, and 11.5% severe pain. Depressive symptoms were present in 8.3% overall (women 9.1%, men 7.5%). Participants with no pain, mild, moderate, and severe pain reported depressive symptoms in 2.5%, 6.5%, 14.4%, and 27.1%, respectively. In the multivariable analysis, higher pain intensity was associated with a higher risk of depressive symptoms (mild pain OR 1.8, 95% CI 1.3–2.4; moderate pain OR 2.8, 95% CI 2.0–4.0; severe pain OR 4.0, 95% CI 2.8–5.6). Depressive symptoms were further associated with a Body Mass Index (BMI) under 18.5 kg m^−2^ (OR 2.4; 95% CI 1.4–4.1), but not with sex (OR 1.1; 95% CI 0.9–1.3).

**Conclusions:**

Higher pain intensity increases the risk of depressive symptoms. We suggest regular assessment of pain and further assessment of depressive symptoms in patients with moderate or severe pain.

**Significance Statement:**

Our study found a clear association between higher pain intensity and depressive symptoms in the general population across all types of pain. Further, being underweight was linked to depressive symptoms overall and the highest prevalence of depressive symptoms was found in underweight persons with severe pain. These findings highlight the importance of assessing depressive symptoms in patients with higher pain intensity, especially in underweight patients.

## 1 INTRODUCTION

Pain is experienced by 7.4%–46.0% of the European population (Häuser et al., 2013; Leadley et al., 2012). According to the Global Burden of Disease (GBD) study 2019 it represents the main characteristic of multiple leading causes of disability‐adjusted life‐years (DALYs) (Abbafati et al., 2020). However, pain is a complex phenomenon that can take an acute or chronic course (Kuner & Kuner, 2021) and can have multiple biopsychosocial consequences (Breivik et al., 2006).

As depression was ranked the 13th leading cause of DALYs by the GBD study 2019 (Abbafati et al., 2020), both pain and depression are respectively the most common physical and psychological symptom‐based disorders (Kroenke et al., 2011). Their co‐occurrence significantly impacts disability (Hall et al., 2011), treatment outcomes (Kroenke et al., 2011), absence from work (Munce et al., 2006), and health‐related quality of life (HRQoL) (Robert Koch‐Institut (Hrsg), 2021; IsHak et al., 2018). Notably, there is a considerable overlap between these conditions. Of the people suffering from depression approximately 65% also experience pain (Bair et al., 2003). People suffering from pain also report depression in 5%–85% (Bair et al., 2003; Breivik et al., 2006). However, the prevalence of this comorbidity varies depending on the study population and type of pain assessed (Bair et al., 2003; Breivik et al., 2006).

Most studies on the co‐occurrence of pain and depression have concentrated on specific patient populations, such as those diagnosed with rheumatoid arthritis (Jobski et al., 2017) or HIV (Hémar et al., 2022), or on specific types of pain including lower back pain (Martini & Hoffmann, 2018), neck and shoulder pain, musculoskeletal problems (Bair et al., 2003), acute and chronic postoperative pain (Ghoneim & O'Hara, 2016), and various other chronic pain conditions (Munce et al., 2007). Chronic pain, in isolation, increases the risk of depression by a factor of 2 to 7.6 (Huang et al., 2021; Miller & Cano, 2009). However, only a few studies assessed the intensity of pain as a risk factor for depression. In a 12‐month follow‐up study of patients experiencing persistent back, hip, or knee pain a link between increasing pain intensity and depression could be seen (Kroenke et al., 2011). In patients with neuropathic pain, depression was shown to have an association with the duration of treatment as well as with pain severity (Cherif et al., 2020). A study conducted in the general Canadian population showed a connection between higher pain intensity and depression. However, this Canadian study only included four chronic pain conditions: self‐disclosed fibromyalgia, arthritis or rheumatism, back problems, and migraine headaches (Munce et al., 2007). Research with comprehensive insights into the general population, including all types of pain, and exploring variations in pain intensity remains scarce. An understanding of the impact on the general population could assist general practitioners in determining when to assess depressive symptoms in patients experiencing pain.

Therefore, our study seeks to investigate the prevalence of any type of pain, depressive symptoms, and their co‐occurrence, differentiated by the levels of pain intensity in the general German population.

## 2 METHODS

### 2.1 Study design and sample

This study utilizes data from the cross‐sectional German Health Update (GEDA). Based on and including the questionnaire of the European Health Interview Survey (EHIS), the GEDA study was carried out by the Robert Koch Institute (RKI) on behalf of the German Federal Ministry of Health (Hintzpeter et al., 2019). Data were collected from April 2019 to September 2020 (Allen et al., 2021). Implemented as a telephone survey, the GEDA 2019/2020‐EHIS utilized a computer‐assisted, fully structured interview method. Random samples of both landline and mobile telephone numbers using a dual frame method were selected (von der Heyde, 2013). The survey targeted people living permanently in Germany aged 15 years and older, with 23,001 interviews sufficiently completed (response 21.6%). The data for scientific use, which was used in our study, includes all participants who were 18 years and older at the time of the survey and totals 22,708 participants (Robert Koch Institute & Department of Epidemiology and Health Monitoring, 2022). Further details on methods and non‐response are described elsewhere (Allen et al., 2021).

### 2.2 Indicators

The GEDA 2019/2020‐EHIS included a wide range of questions regarding health status, healthcare provision, health determinants, and socioeconomic factors.

As an indicator of pain, participants were asked: ‘How intense was your pain in the last 4 weeks?’. The response options included ‘no pain’, ‘very mild’, ‘mild’, ‘moderate’, ‘severe’, and ‘very severe’. In the literature, it is customary to categorize pain intensity into ‘no pain’, ‘mild’, ‘moderate’, and ‘severe’ (Breivik et al., 2008). Thus, the response options ‘very mild’ and ‘mild’ were combined into the category ‘mild’, and the response options ‘severe’ and ‘very severe’ were combined into the category ‘severe’. For current depressive symptoms, the internationally established 8‐item Patient Health Questionnaire (PHQ‐8) was used. The PHQ‐8 is a brief measure for depressive symptoms (Kroenke et al., 2009) where persons are asked about the frequency of eight specific depressive symptoms in the last two weeks. The PHQ‐8's total score ranges from 0 to 24 and the presence of depressive symptoms is assumed when the score is ≥10 (Kroenke et al., 2001).

Furthermore, the following covariables were evaluated. For sociodemographic variables sex assigned at birth (women, men), age (18–29, 30–44, 45–64, 65–79, 80+ years), and education level (International Standard Classification of Education, ISCED: low, medium, and high education group) were used (UNESCO Institute for Statistics, 2012). Self‐assessed health status included the World Health Organization's (WHO) recommended question ‘How is your health in general?’, which is also included in the internationally established 36‐Item Short Form Survey Instrument (SF‐36) (Brazier et al., 1992). The response options were ‘very good’, ‘good’, ‘fair’, ‘bad’, ‘very bad’. The responses ‘very good’ or ‘good’ can be defined as a positively perceived subjective health (Heidemann et al., 2021). Further, the presence of a chronic disease or long‐term health problem was recorded via the question: ‘Do you have any chronic disease or a long‐term health problem? This means diseases or health problems that have lasted or are expected to last for at least 6 months’ with the response options ‘yes’ or ‘no’. Furthermore, the body mass index (BMI) was analysed and categorized according to the WHO into underweight (<18.5 kg m^−2^), normal body weight (18.5 to <25 kg m^−2^), overweight (25–30 kg m^−2^), and obesity (above 30 kg m^−2^) (World Helath Organization, n.d.).

### 2.3 Statistical analyses

The prevalence of each pain intensity was described in percentages with a 95% confidence interval (95% CI) and stratified by all covariables. The prevalence of depressive symptoms was further analysed for each pain intensity level, stratified by each covariable. The denominator may vary because participants who did not provide information for the specific variable were excluded from that analysis.

We conducted a multivariable logistic regression analysis to assess the influence of pain intensity on depressive symptoms adjusting for all covariables. The results were presented as odds ratio (OR) with a 95% CI.

Additionally, we performed a subgroup analysis including only participants with a positively perceived subjective health thereby excluding participants with assumedly more complex health problems.

To correct the deviations of the sample compared to the German population a weighting factor was used, according to age, sex, federal state and district type (as of 31 December 2019), and education level based on the microcensus 2017 (Allen et al., 2021; Research Data Centres of the Federal Statistical Office and Statistical Offices of the Länder, 2017).

Data analyses were carried out using IBM SPSS Statistics for Macintosh, Version 29.0. To account for the weighting factor the complex sampling function was used. A statistically significant difference between groups is assumed if the 95% CI of the prevalences do not overlap or the 95% CI of the OR does not include 1, respectively.

An ethics vote for the GEDA 2019/2020‐EHIS study was obtained from the Ethics Committee of the Charité University Medicine, Berlin (EA2/070/19) (Allen et al., 2021).

## 3 RESULTS

### 3.1 Characteristics of the study population

The characteristics of the study sample are presented in Table 1. The analyses included data from 22,708 participants and 51% were women. Most participants were between 45 and 64 years old (35.1%), followed by 30–44 years (22.7%). Most participants had a medium education level (57.1%) and good subjective health (46.0%), and a normal body weight (44.2%), 34.4% were overweight. Out of all participants, 49.2% had a chronic disease or health problem for at least 6 months.

**TABLE 1:** Baseline characteristics, in % (weighted) with unweighted count.

	Overall (*n* = 22,708), unweighted
	% (*n*, unweighted)
Sex	
Women	51.0 (*n* = 11,968)
Men	49.0 (*n* = 10,740)
Age groups	
18–29 years	16.3 (*n* = 2101)
30–44 years	22.7 (*n* = 3769)
45–64 years	35.1 (*n* = 8981)
65–79 years	18.0 (*n* = 6048)
80+ years	8.0 (*n* = 1809)
Education level	
Low	17.8 (*n* = 1339)
Medium	57.1 (*n* = 9670)
High	25.2 (*n* = 11,637)
Subjective health	
Very good	23.9 (*n* = 5514)
Good	46.0 (n = 11,075)
Fair	22.2 (*n* = 4665)
Bad	6.4 (*n* = 1191)
Very bad	1.5 (*n* = 251)
Chronic disease or health problem	49.2 (*n* = 11,481)
BMI	
< 18.5 kg m^−2^	2.3 (*n* = 420)
18.5 – <25 kg m^−2^	44.2 (*n* = 10,025)
25 to <30 kg m^−2^	34.4 (*n* = 8094)
≥ 30 kg m^−2^	19.0 (*n* = 3875)

Abbreviations: BMI, body mass index; CI, confidence Interval; kg, kilogram; m, metre.

### 3.2 Prevalence of pain intensity categories

Table 2 shows that 41.2% of participants reported no pain, 32.1% mild pain, 15.3% moderate pain, and 11.5% severe pain in the last 4 weeks. Women reported higher pain intensity more often than men, having severe pain in 13.5% and moderate pain in 18.0%, compared to 9.4% and 12.4% of men. The prevalence of higher pain intensity increased with age. Out of the participants aged 80 years and older 19% reported severe pain, while only 4.5% of participants aged 18–29 years did the same. Those with a low education level reported severe pain in 17.8% and those with a high education level in 7.1%. Worse subjective health was also linked to higher pain levels. For instance, 69.0% of participants with very bad and 49.1% with bad subjective health reported severe pain, compared to 2.8% of those with very good subjective health. Participants suffering from chronic diseases or health problems for at least 6 months reported higher pain levels more often, compared to those without such conditions. The prevalence of severe pain by BMI level revealed a comparatively small variation from 8.7% (participants with a BMI of 18.5 – <25 kg m^−2^) to 17.7% (those with a BMI of ≥30 kg m^−2^).

**TABLE 2:** Distribution of pain intensity overall and stratified, in % (weighted) with 95% CI.

	Pain intensity in the last four weeks			
	No pain *n* = 9474	Mild pain *n* = 7523	Moderate pain *n* = 3362	Severe pain *n* = 2330
	% (95% CI)	% (95% CI)	% (95% CI)	% (95% CI)
Overall (*n* = 22,689)	41.2 (40.2–42.2)	32.1 (31.2–33.0)	15.3 (14.6–16.0)	11.5 (10.8–12.2)
Sex (*n* = 22,689)				
Women	36.4 (35.1–37.7)	32.2 (31.0–33.5)	18.0 (16.9–19.0)	13.5 (12.5–14.5)
Men	46.2 (44.7–47.6)	31.9 (30.6–33.3)	12.4 (11.5–13.4)	9.4 (8.6–10.4)
Age groups (*n* = 22,689)				
18–29 years	48.6 (45.8–51.4)	36.9 (34.2–39.7)	10.0 (8.4–11.9)	4.5 (3.4–5.9)
30–44 years	48.7 (46.5–51.0)	31.1 (29.1–33.2)	12.2 (10.7–13.7)	8.0 (6.7–9.4)
45–64 years	37.1 (35.6–38.5)	31.6 (30.2–33.0)	17.3 (16.1–18.5)	14.0 (12.9–15.3)
65–79 years	37.8 (36.0–39.8)	30.2 (28.4–32.0)	18.0 (16.4–19.6)	14.0 (12.6–15.5)
80+ years	30.2 (27.0–33.7)	31.2 (28.0–34.5)	19.6 (16.9–22.7)	19.0 (16.1–22.1)
Education level (*n* = 22,677)				
Low	37.1 (34.0–40.3)	27.7 (25.0–30.6)	17.3 (15.1–19.9)	17.8 (15.5–20.4)
Medium	39.6 (38.3–40.9)	32.8 (31.5–34.0)	16.2 (15.2–17.2)	11.4 (10.6–12.3)
High	47.5 (46.3–48.7)	33.5 (32.4–34.7)	11.9 (11.1–12.7)	7.1 (6.5–7.7)
Subjective health (*n* = 22,689)				
Very good	69.3 (67.5–71.1)	24.2 (22.6–25.9)	3.7 (3.0–4.4)	2.8 (2.1–3.7)
Good	43.3 (41.9–44.7)	38.6 (37.3–40.0)	13.0 (12.1–14.1)	5.0 (4.4–5.7)
Fair	17.6 (15.9–19.4)	33.0 (30.9–35.1)	29.8 (27.9–31.8)	19.7 (18.0–21.5)
Bad	10.3 (8.1–13.1)	15.9 (13.1–19.3)	24.7 (21.1–28.6)	49.1 (44.8–53.4)
Very bad	7.5 (4.4–12.4)	10.9 (6.4–18.1)	12.6 (7.8–19.6)	69.0 (60.3–76.5)
Chronic disease or health problem (*n* = 22,620)				
Yes	25.8 (24.6–27.1)	32.4 (31.1–33.8)	22.4 (21.2–23.5)	19.4 (18.2–20.6)
No	56.1 (54.7–57.5)	31.7 (30.4–33.0)	8.3 (7.5–9.2)	3.9 (3.3–4.5)
BMI (*n* = 22,398)				
< 18.5 kg m^−2^	37.9 (31.3–45.0)	34.3 (27.6–41.8)	14.1 (10.1–19.3)	13.7 (9.1–20.1)
18.5 to <25 kg m^−2^	45.9 (44.4–47.4)	32.2 (30.8–33.6)	13.1 (12.2–14.2)	8.7 (7.9–9.7)
25 to <30 kg m^−2^	41.6 (40.0–43.2)	31.9 (30.4–33.4)	15.0 (13.8–16.2)	11.5 (10.5–12.7)
≥ 30 kg m^−2^	29.8 (27.7–32.0)	31.9 (29.7–34.1)	20.6 (18.7–22.6)	17.7 (15.9–19.6)

*Note*: *n* refers to the (unweighted) number of persons. Pain intensity refers to the last 4 weeks.

Abbreviations: BMI, body mass index; CI, confidence Interval; kg, kilogram; m, metre.

### 3.3 Depressive symptoms in relation to pain intensity

As displayed in Table 3, 8.3% of participants reported depressive symptoms in the last 2 weeks. The prevalence of depressive symptoms increased with higher pain intensity. Depressive symptoms were reported by 2.5% of participants with no pain, 6.5% with mild pain, 14.4% with moderate pain, and 27.1% with severe pain. Overall, women suffered from depressive symptoms in 9.1%, and men in 7.5%. Both sexes showed an almost equal prevalence of depressive symptoms throughout the individual pain intensity levels, with a steady increase at higher pain intensity. A higher proportion of younger people (18–29 years, 30–44 years, and 45–64 years) suffered from depressive symptoms (9.7%, 8.1%, and 9.9%, respectively) compared to older participants (65–79 years: 4.8%; 80+ years: 6.7%). This was seen at all pain intensity levels. In each age group, the prevalence of depressive symptoms increased with higher pain intensity. Overall and at all pain intensity levels, the prevalence of depressive symptoms steadily decreased with higher education levels. Participants who reported very bad or bad subjective health had significantly more often depressive symptoms (46.1% and 39.8%, respectively) than those reporting better subjective health (ranging from 1.4% to 14.3%). Participants with very good and good subjective health had a higher increase in the prevalence of depressive symptoms than participants with fair or bad subjective health whereas participants with very bad subjective health had no clear dynamic in the prevalence of depressive symptoms at the different pain intensity levels. Individuals suffering from chronic diseases or health problems reported a higher prevalence of depressive symptoms (13.7%) than those without such conditions (3.1%). This was seen at all pain intensity levels with both groups having an increasing prevalence of depressive symptoms with higher pain intensity. Within the BMI categories, participants with a BMI under 18.5 kg m^−2^ exhibited the highest prevalence of depressive symptoms at 22.7%, followed by those with a BMI of 30 kg m^−2^ and higher (11.5%). While all BMI groups had an increase in depressive symptoms with higher pain intensity, participants with a BMI under 18.5 kg m^−2^ had the highest prevalence of depressive symptoms at all pain intensity levels (except in moderate pain). The other three BMI groups had a quite similar prevalence of depressive symptoms at each pain intensity level.

**TABLE 3:** Prevalence of depressive symptoms stratified by pain intensity and overall, in % (weighted) with 95% CI.

	Overall *n* = 22,250	Pain intensity in the last four weeks			
		No pain *n* = 9359	Mild pain *n* = 7389	Moderate pain *n* = 3274	Severe pain *n* = 2228
Depressive symptoms	% (95% CI)	% (95% CI)	% (95% CI)	% (95% CI)	% (95% CI)
Overall (*n* = 22,250)	8.3 (7.7–9.0)	2.5 (2.0–3.1)	6.5 (5.5–7.6)	14.4 (12.4–16.7)	27.1 (24.1–30.2)
Sex (*n* = 22,250)					
Women	9.1 (8.2–10.0)	2.5 (1.8–3.5)	6.5 (5.3–8.1)	14.4 (12.0–17.2)	26.5 (22.9–30.5)
Men	7.5 (6.6–8.5)	2.4 (1.8–3.3)	6.4 (4.9–8.2)	14.4 (11.2–18.3)	27.8 (23.1–33.1)
Age groups (*n* = 22,250)					
18–29 years	9.7 (7.9–11.8)	3.9 (2.6–5.8)	11.9 (8.7–16.0)	23.7 (15.8–34.1)	23.1 (13.2–37.2)
30–44 years	8.1 (6.8–9.7)	2.6 (1.6–4.1)	6.9 (4.9–9.6)	16.8 (12.4–22.3)	34.6 (25.9–44.5)
45–64 years	9.9 (8.8–11.1)	2.5 (1.7–3.7)	5.6 (4.3–7.4)	14.7 (11.8–18.1)	34.6 (29.9–39.6)
65–79 years	4.8 (3.9–5.8)	0.9 (0.5–1.6)	3.1 (1.8–5.2)	7.4 (4.9–11.0)	15.8 (12.1–20.4)
80+ years	6.7 (5.0–8.9)	1.3 (0.5–3.3)	3.0 (1.6–5.5)	13.8 (8.2–22.2)	14.3 (9.3–21.4)
Education level (*n* = 22,198)					
Low	13.7 (11.5–16.3)	4.6 (2.8–7.4)	12.0 (8.3–17.0)	20.9 (15.0–28.5)	29.7 (22.8–37.7)
Medium	8.4 (7.6–9.3)	2.5 (1.9–3.4)	6.3 (5.0–7.8)	13.8 (11.5–16.6)	28.1 (24.4–32.1)
High	4.4 (3.9–4.9)	1.2 (0.9–1.6)	3.8 (3.0–4.8)	9.9 (7.7–12.6)	19.8 (16.6–23.4)
Subjective health (*n* = 22,242)					
Very good	1.4 (1.0–2.1)	0.7 (0.4–1.3)	2.3 (1.2–4.1)	2.2 (0.6–7.6)	10.6 (4.0–25.1)
Good	3.8 (3.2–4.5)	1.8 (1.3–2.5)	3.8 (2.8–5.1)	9.3 (6.7–12.7)	7.4 (4.5–12.1)
Fair	14.3 (12.6–16.2)	9.3 (6.0–14.1)	12.2 (9.5–15.6)	14.1 (11.3–17.4)	22.5 (18.2–27.5)
Bad	39.8 (35.3–44.4)	21.5 (11.7–36.2)	34.1 (23.9–46.0)	38.3 (29.6–47.7)	46.4 (40.0–52.9)
Very bad	46.1 (36.7–55.8)	52.9 (27.8–76.7)	24.9 (7.2–58.6)	54.6 (30.6–76.6)	47.2 (35.6–59.1)
Chronic disease or health problem (*n* = 22,189)					
Yes	13.7 (12.6–14.9)	5.3 (4.0–7.0)	9.8 (8.1–11.9)	15.8 (13.5–18.4)	29.6 (26.4–33.1)
No	3.1 (2.6–3.8)	1.2 (0.8–1.7)	3.2 (2.3–4.3)	11.0 (7.4–16.0)	14.8 (9.3–22.8)
BMI (*n* = 21,985)					
< 18.5 kg m^−2^	22.7 (16.3–30.7)	13.9 (7.5–24.1)	26.6 (15.4–42.0)	9.1 (2.9–25.3)	51.5 (30.4–72.1)
18.5 – <25 kg m^−2^	7.1 (6.2–8.1)	1.9 (1.3–2.8)	6.2 (4.8–8.0)	13.8 (10.7–17.7)	28.9 (23.5–35.1)
25 – <30 kg m^−2^	7.0 (6.1–8.1)	2.3 (1.5–3.5)	4.3 (3.1–5.8)	13.7 (10.5–17.7)	23.7 (19.2–28.7)
≥ 30 kg m^−2^	11.5 (10.0–13.3)	2.7 (1.5–4.8)	8.4 (5.9–11.7)	15.9 (12.3–20.5)	27.6 (22.7–33.1)

*Note*: *n* refers to the (unweighted) number of persons. Pain intensity refers to the last 4 weeks. Depressive symptoms refer to the last 2 weeks.

Abbreviations: BMI, body mass index; CI, confidence Interval; kg, kilogram; m, metre.

### 3.4 Associated factors of depressive symptoms

**FIGURE 1:**
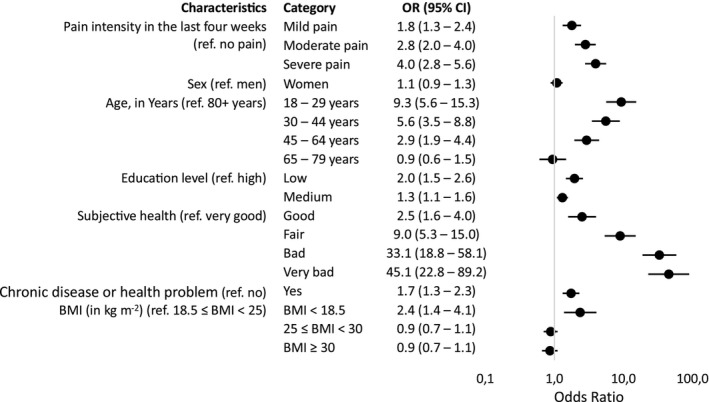
Multivariable logistic regression analysis: Associated factors of depressive symptoms (*n* = 21,872). *n* refers to the (unweighted) number of persons. Pain intensity refers to the last 4 weeks. Depressive symptoms refer to the last 2 weeks. CI, Confidence Interval; ref, Reference; BMI, Body Mass Index; kg, kilogram; m, metre.

Most of the factors associated with depressive symptoms identified in the stratified analyses were confirmed in the multivariable logistic regression (Figure 1). Higher pain intensity was associated with more depressive symptoms. The impact increased as the pain intensity increased (OR for mild pain: 1.8; OR for moderate pain: 2.8; OR for severe pain: 4.0). Furthermore, depressive symptoms were linked to being under 64 years old, a lower education level, having a chronic disease or health problem for at least 6 months (OR: 1.7), a BMI under 18.5 kg m^−2^ (OR: 2.4), and poorer subjective health. Very bad subjective health had the highest impact (OR: 45.1) on depressive symptoms, followed by bad subjective health (OR: 33.1), and the age between 18 and 29 (OR: 9.3).

### 3.5 Subgroup analysis of participants with positively perceived subjective health

Table S1 shows the prevalence of depressive symptoms overall and stratified by pain intensity of participants with a positively perceived subjective health. Data from 16,375 participants was included (72.1% of the overall population). The prevalence of depressive symptoms increased with higher pain intensity from 1.3% (no pain) to 3.4% (mild pain), reaching a plateau of more than 8% (moderate and severe pain).

The multivariable logistic regression (*n* = 16,135; Figure S1) showed similar associated factors compared to the overall group. Higher pain intensity was associated with a higher risk of depressive symptoms, with an OR of 2.1 for mild, 5.3 for moderate and 5.6 for severe pain. The age group between 18 and 29 showed the highest impact on depressive symptoms (OR: 8.0), followed by severe pain (OR: 5.6).

## 4 DISCUSSION

In our population‐based study, the prevalence of depressive symptoms increased significantly with higher pain intensity. While women had a higher overall prevalence of depressive symptoms, there was no sex difference when taking pain intensity into account. Participants with a BMI under 18.5 kg m^−2^ showed the highest prevalence of depressive symptoms within the BMI groups.

### 4.1 Co‐occurring pain and depressive symptoms

The prevalence of moderate and severe pain in our study is comparable to the 12.0%–30.0% reported by a European study (Breivik et al., 2006). Our study found an 8.3% prevalence of depressive symptoms, falling within the range reported for the European Union (2.7%–10.0%) (Hapke et al., 2019).

Suffering from pain increased the prevalence of depressive symptoms in our study. This is consistent with the literature, which shows chronic pain and lower back pain to elevate the risk of depression (Huang et al., 2021; Martini & Hoffmann, 2018; Miller & Cano, 2009). Additionally, pain and depression often co‐occur in rheumatoid arthritis patients (Jobski et al., 2017). A family‐based cohort and twin study demonstrated a higher prevalence of pain and depression co‐occurring, even after adjusting for socioeconomic and environmental factors (Van Hecke et al., 2017), agreeing with our regression analysis.

In our study, increasing pain intensity was associated with a considerably higher prevalence of depressive symptoms. Few studies have assessed pain intensity in connection with depressive symptoms. When assessed, most studies focused on specific pain types or populations (Bair et al., 2003; Kroenke et al., 2011; Munce et al., 2007). A systematic review of studies including patients in different medical clinics, such as psychiatry, orthopaedic, and primary care, looking at specific types of pain revealed that as pain factors—Including intensity—Worsen, the occurrence of depression increases (Bair et al., 2003). This mutual negative effect was also seen in a 12‐month prospective study of patients with persistent back, hip, or knee pain (Kroenke et al., 2011). Our findings are comparable with a study examining chronic pain conditions in the general Canadian population. However, the prevalence of depression in participants with mild pain (8.3% men; 13.8% women) was higher, and with severe pain (19.6% men; 23.2% women) lower in the Canadian study. This may be due to Munce and colleagues focusing on chronic pain and major depressive disorder (Munce et al., 2007). Our study included all types of pain and analysed depressive symptoms independent of severity and, therefore, might be more representative of the general population. Highlighting this finding in the general population is important because depression often remains underdiagnosed (Bair et al., 2003). Pain and pain intensity are also frequently underestimated by healthcare professionals (Seers et al., 2018). Depression and pain can lead to less recognition of each other (Bair et al., 2003) and can decrease each other's treatment response (IsHak et al., 2018). Consequently, an accurate assessment is needed to determine the appropriate treatment. Our analyses among participants with positively perceived subjective health showed a significantly increased depressive symptoms prevalence from mild to moderate pain. Conversely, no such linear relationship was observed in those with fair, bad or very bad subjective health at this cut‐off. This is likely because this group of patients experiences complex health problems more often, where pain might only play a small role.

Our findings indicate, that even in people with good subjective health higher pain intensity significantly increases the risk of depressive symptoms, suggesting moderate pain as a useful cut‐off for assessing depressive symptoms. We, therefore, recommend regular pain assessment in general care settings and further assessment of depressive symptoms in patients with moderate or severe pain.

### 4.2 The influence of sex on co‐occurring pain and depressive symptoms

Women showed a higher depressive symptoms prevalence overall, but we saw no sex difference when taking pain intensity into account. Depressive symptoms were reported by 9.1% of women and 7.5% of men, aligning with EU ranges (3.4%–12.9% for women; 2.0%–8.2% for men), showing a higher prevalence in women (Hapke et al., 2019).

Research on sex's influence on co‐occurring pain and depression is inconclusive. Our study found no significant sex difference after adjusting for pain intensity, consistent with a general population study in Michigan, USA (Miller & Cano, 2009). Several studies suggest women are more likely to experience chronic pain and co‐occurring depression (Haley et al., 1985; Martini & Hoffmann, 2018; Munce et al., 2007; Tsang et al., 2008). In 55‐85‐year‐olds, men exhibited a stronger pain‐depression association (Geerlings et al., 2002). These studies, including the aforementioned Canadian study, analysed chronic pain conditions and mainly assessed major depression.

Our study indicated an association between higher pain intensity and depressive symptoms. Against this background, the higher overall depressive symptoms prevalence among women in our study may be due to women reporting higher pain intensity more often. The literature describes a higher pain prevalence, lower pain threshold and tolerance in women (Bartley & Fillingim, 2013; Mills et al., 2019). Oestrogens may contribute to women's higher levels of pain by modulating the nervous and other body systems (Craft, 2007). Biological factors, like stress responsiveness or limbic system hyperactivity, and psychosocial stressors could also contribute to the sex difference in depression (Bangasser & Cuarenta, 2021; Parker & Brotchie, 2010). Further research should assess sex differences in depressive symptoms at different pain intensity levels.

### 4.3 The influence of bodyweight on co‐occurring pain and depressive symptoms

Underweight participants had the highest prevalence of depressive symptoms (22.7%). This compares to the 24% prevalence of depression in underweight primary care patients in Australia (Carey et al., 2014). While the literature links both underweight and obesity to depression (Carey et al., 2014; Mills et al., 2019), our regression showed this association only for underweight. Despite the small number of underweight participants in our study, the results are noteworthy. One could argue that the prevalence of depressive symptoms is linked to being underweight solely due to the association between underweight and lower subjective health (Tang et al., 2017), which in turn is linked to depressive symptoms (Ghaemi Kerahrodi et al., 2019). Notwithstanding, being underweight was also associated with depressive symptoms in participants with positively perceived subjective health. The literature analysing the association between underweight and depression in community‐based settings is inconclusive (De Wit et al., 2009; Kelly et al., 2011). Many studies focused on eating disorders, which may limit the representation of people with low BMI (Kelly et al., 2011).

In our study, 51.5% of underweight participants with severe pain reported depressive symptoms. Previous studies have shown an association of depression with greater pain experience in patients with eating disorders (Fazia et al., 2023). Therefore, pain and depressive symptoms assessment should be considered, particularly in underweight patients with either condition. Although depression may be a potential link between pain and underweight, the interconnection of these three conditions is not yet fully understood, making further research necessary.

### 4.4 Strengths and limitations

The major strength of this study is the large number of participants from the general German population. To our knowledge, it is the first study analysing the general population while including all types of pain and different pain intensity levels. The indicator for depressive symptoms was not restricted to major depression but used the internationally established PHQ‐8 (Kroenke et al., 2009), hereby addressing the problem of underdiagnosed depression (Bair et al., 2003).

However, this study has several limitations due to the underlying data of the GEDA 2019/2020‐EHIS survey (Allen et al., 2021). The study's cross‐sectional design did not allow to determine a causal relationship between depressive symptoms and higher pain intensity. The same applies to (low) BMI and the presence of depressive symptoms. The reported pain intensity could be biased since research suggests memory of painful events is not always accurate and affected by various factors (Breivik et al., 2008). Due to the original study design pain entities could not be differentiated and, because participants were asked about any pain in the last 4 weeks, no differentiation between acute and chronic pain was possible either. Also, the indicators refer to various time frames. The GEDA 2019/2020‐EHIS survey was partly conducted during the first wave of the COVID‐19 pandemic (Tolksdorf et al., 2021), which may have changed people's accessibility and willingness to participate and the prevalence of depressive symptoms. Nonetheless, initial analyses showed no selection bias between the data before and after the COVID‐19 pandemic onset (Heidemann et al., 2021). Because of possible non‐response, certain characteristics may deviate. This was counteracted by weighting for age, sex, region, and education level.

## 5 CONCLUSION

Our study found an almost linear association between higher pain intensity and depressive symptoms prevalence, showing no sex difference. Being underweight was linked to depressive symptoms overall. The highest prevalence of depressive symptoms was found in underweight persons with severe pain. As pain and depression are frequently underdiagnosed, we recommend regular pain assessment in general care settings and further assessment of depressive symptoms in patients with moderate or severe pain, especially in underweight patients. Further research should assess the longitudinal association of acute and chronic pain intensity and depressive symptoms in general population settings.

## AUTHOR CONTRIBUTIONS

FH, KJ and JNA conceived the idea for this article. JNA processed, analysed, and interpreted the data, which was validated by FH and KJ. CB provided clinical expertise. JNA drafted the manuscript. FH, KJ, and CB critically reviewed the manuscript. All authors reviewed and approved the final manuscript.

## FUNDING INFORMATION

The GEDA 2019/2020‐EHIS study was funded by the Robert Koch Institute and the German Federal Ministry of Health. The Robert Koch Institute is a Federal Institute within the Federal Ministry of Health. The present study did not receive additional funding.

## CONFLICT OF INTEREST STATEMENT

The authors declare no conflict of interest.

## Supporting information


Figure S1.

Table S1.
