# Supplementary material for: Pain intensity and comorbid depressive symptoms in the general population: An analysis of the German Health Update Study (GEDA 2019/2020‐EHIS)
Source: Eur J Pain. 2024 Oct 23;29(3):e4745. doi: 10.1002/ejp.4745 (PMC11755701; doi:10.1002/ejp.4745)
Supplement: Supplementary file 1 — Figure S1. [file EJP-29-0-s001.pdf]

Supplementary Figures

Characteristics	Category	OR (95% CI)
Pain intensity in the last four weeks (ref. no pain)	Mild pain	2.1 (1.4 - 3.2)
	Moderate pain	5.3 (3.3 - 8.6)
	Severe pain	5.6 (3.0 - 10.5)
Sex (ref. men)	Women	0.9 (0.7 - 1.3)
Age, in Years (ref. 80+ years)	18 – 29 years	8.0 (2.6 - 24.8)
	30 – 44 years	3.7 (1.2 - 11.4)
	45 – 64 years	2.0 (0.7 - 6.0)
	65 – 79 years	0.4 (0.1 - 1.4)
Education level (ref. high)	Low	2.8 (1.7 - 4.5)
	Medium	1.6 (1.1 - 2.2)
Subjective health (ref. very good)	Good	2.2 (1.4 - 3.7)
Chronic disease or health problem (ref. no)	Yes	1.9 (1.3 - 2.7)
BMI (in kg m^{-2}) (ref. 18.5 ≤ BMI < 25)	BMI < 18.5	2.7 (1.2 - 6.1)
	25 ≤ BMI < 30	1.0 (0.7 - 1.5)
	BMI ≥ 30	1.0 (0.6 - 1.6)

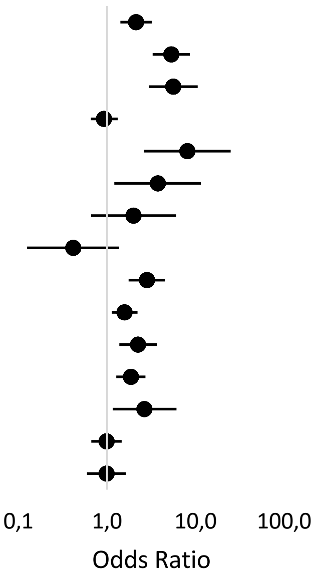

Supplementary Fig. 1: Multivariable logistic regression analysis: Associated factors of depressive symptoms, a subpopulation with very good or good subjective health (n=16,135).

Legend Supplementary Fig. 1:
n refers to the (unweighted) number of persons. Pain intensity refers to the last four weeks. Depressive symptoms refer to the last two weeks CI = Confidence Interval; ref. = Reference; BMI = Body Mass Index; kg = kilogram; m = metre
