# Supplementary material for: Pain intensity and comorbid depressive symptoms in the general population: An analysis of the German Health Update Study (GEDA 2019/2020‐EHIS)
Source: Eur J Pain. 2024 Oct 23;29(3):e4745. doi: 10.1002/ejp.4745 (PMC11755701; doi:10.1002/ejp.4745)
Supplement: Supplementary file 2 — Table S1. [file EJP-29-0-s002.pdf]

Supplementary Tables

Supplementary Table 1: Prevalence of depressive symptoms stratified by pain intensity and overall, in % (weighted) with 95% CI in participants with positively perceived subjective health

		Pain intensity in the last four weeks			
	**Overall** n=16375	**No pain** n=8458	**Mild pain** n=5682	**Moderate pain** n=1555	**Severe pain** n=680
**Depressive Symptoms**	% (95% CI)	% (95% CI)	% (95% CI)	% (95% CI)	% (95% CI)
Overall (n=16,375)	3.0 (2.6 – 3.5)	1.3 (1.0 – 1.7)	3.4 (2.6 – 4.4)	8.4 (6.1 – 11.4)	8.1 (5.2 – 12.5)
Sex (n=16,375)					
Women	3.2 (2.6 – 3.9)	1.6 (1.1 – 2.3)	3.1 (2.2 – 4.3)	8.6 (5.9 – 12.4)	6.6 (3.8 – 11.0
Men	2.8 (2.2 – 3.6)	1.1 (0.7 – 1.7)	3.8 (2.6 – 5.6)	8.1 (4.7 – 13.7)	10.3 (5.0 – 19.8)
Age groups (n=16,375)					
18-29 years	6.4 (4.9 – 8.3)	2.9 (1.9 – 4.4)	7.8 (5.2 – 11.6)	20.6 (12.1 – 32.7)	14.2 (5.6 – 31.5)
30-44 years	3.1 (2.3 – 4.1)	1.2 (0.7 – 2.2)	3.2 (2.0 – 5.0)	11.1 (6.6 – 18.0)	10.8 (4.5 – 23.6)
45-64 years	2.2 (1.7 – 2.8)	0.9 (0.5 – 1.5)	2.3 (1.5 – 3.6)	4.5 (2.6 – 7.7)	8.5 (4.3 – 16.0)
65-79 years	0.5 (0.3 – 0.8)	0.5 (0.2 – 1.1)	0.3 (0.1 – 0.7)	1.0 (0.4 – 2.6)	0.3 (0.0 – 1.8)
80+ years	1.5 (0.5 – 4.0)	0.6 (0.1 – 4.0)	1.1 (0.2 – 7.6)	5.8 (1.1 – 26.0)	1.5 (0.4 – 6.4)
Education level (n=16,333)					
Low	5.2 (3.6 – 7.4)	2.8 (1.26– 5.0)	7.4 (4.0 – 13.1)	10.7 (4.7 – 22.9)	5.3 (1.5 – 17.4)
Medium	3.1 (2.6 – 3.9)	1.2 (0.8 – 1.8)	3.2 (2.2 – 4.6)	9.6 (6.6 – 13.8)	10.8 (6.1 – 18.5)
High	1.7 (1.3 – 2.1)	0.7 (0.5 - 1.0)	2.3 (1.6 – 3.3)	3.8 (2.4 – 5.9)	5.3 (2.8 – 9.7)
Chronic disease or health problem (n=16,340)					
Yes	4.6 (3.8 – 5.7)	2.4 (1.6 – 3.5)	4.6 (3.1 – 6.7)	7.8 (5.4 – 11.2)	10.3 (5.8 – 17.5)
No	2.1 (1.7 – 2.7)	1.0 (0.6 - 1.4)	2.7 (1.9 – 3.8)	9.2 (5.5 – 15.1)	5.2 (2.6 – 10.2)
BMI (n=16,206)					
<18.5 kg m^{-2}	8.7 (4.7 – 15.5)	8.7 (3.7 – 19.2)	11.6 (4.5 – 26.6)	NA	8.5 (1.2 – 42.3)
18.5 – <25 kg m^{-2}	2.9 (2.3 – 3.7)	1.1 (0.7 – 176)	3.9 (2.7 – 5.6)	9.3 (6.0 – 14.0)	6.9 (3.3 – 13.8)
25 – <30 kg m^{-2}	2.6 (1.9 – 3.4)	1.2 (0.7 – 1.9)	2.5 (1.5 – 4.2)	6.5 (3.4 – 12.0)	11.3 (5.6 – 21.5)
≥30 kg m^{-2}	3.5 (2.3 – 5.1)	1.4 (0.7 – 2.7)	3.0 (1.5 – 5.9)	10.7 (5.4 – 20.2)	5.9 (2.1 – 15.4)

Note: n refers to the (unweighted) number of persons. Pain intensity refers to the last four weeks. Depressive symptoms refer to the last two weeks. CI = Confidence Interval; BMI = Body Mass Index; kg = kilogram; m = metre
